# Nasopharyngeal colonization with pathobionts is associated with susceptibility to respiratory illnesses in young children

**DOI:** 10.1371/journal.pone.0243942

**Published:** 2020-12-11

**Authors:** Timothy J. Chapman, Matthew C. Morris, Lei Xu, Michael E. Pichichero

**Affiliations:** 1 Center for Infectious Diseases and Immunology, Rochester General Hospital Research Institute, Rochester, NY, United States of America; 2 Center for Clinical Systems Biology, Rochester General Hospital Medical Office Building, Rochester, NY, United States of America; University of South Dakota, UNITED STATES

## Abstract

Some children are more susceptible to viral and bacterial respiratory infections in the first few years of life than others. However, the factors contributing to this susceptibility are incompletely understood. In a retrospective analysis of clinical samples collected from a prospectively-enrolled cohort of 358 children we sought associations between physician-attended illness visits and bacterial colonization in the first five years of life. A subset of children was identified by unsupervised clustering analysis as infection and allergy prone (IAP). Several respiratory infection- and allergy-mediated illnesses co-occurred at higher rates in IAP children, while the rates of other illnesses were not significantly different between the groups. Analyses of nasopharyngeal (NP) pathobionts and microbiota commensals showed that early age of first colonization with pathobionts *Streptococcus pneumonia*, non-typeable *Haemophilus influenzae*, and *Moraxella catarrhalis* was associated with IAP children, and particularly *Moraxella* abundance was negatively associated with NP microbiome diversity. We conclude that mucosal pathobiont exposures in early life can influence susceptibility to respiratory illnesses in children.

## Introduction

Infections are common in young children as immune and microbiome development occur. However, some children are more susceptible to infections than others, and the reasons for this are incompletely understood. Postnatal establishment of commensal microbes in the respiratory tract makes an important contribution to immune development and likely influences postnatal respiratory tract morphogenesis [1]. Perturbations in the composition and abundance of respiratory microbiota are associated with a growing number of health conditions [2]. In addition to commensalism, early-life respiratory infections may impact immune development in early life. This has been highlighted by studies linking respiratory infections caused by rhinovirus or RSV and/or colonization with pathogenic bacteria to asthma susceptibility later in childhood [3, 4]. These findings suggest that microbiota commensal composition and pathogenic infections in early life can have a long-lasting influence on health and disease.

The importance of early life exposure to pathobionts, pathogenic bacteria that first colonize as commensals, has been well studied in the case of acute otitis media (AOM) in children, where nasopharyngeal (NP) colonization with *Streptococcus pneumoniae* (*S*. *pneumoniae*), non-typeable *Haemophilus influenzae* (*H*. *influenzae*), or *Moraxella catarrhalis* (*M*. *catarrhalis*) and priming by viral URI are pre-requisites for bacterial invasion of the middle ear [5]. These same pathobionts can additionally cause pneumonia and sinusitis and are associated with asthma incidence, among others. Similar to the overall NP microbiota, environmental factors such as geography, presence of siblings in the home, and daycare attendance can impact pathobiont colonization rates [6–8].

These findings suggest that exposure to respiratory microbiota in early life can influence the incidence of infectious disease and asthma. However, it is unclear how the timing of colonization and abundance of various microbiota within the NP microbiome influence infectious disease outcomes. To address this knowledge gap, we performed a retrospective analysis of clinical samples collected from 6 months to 5 years of age from a prospectively-enrolled cohort of children to determine associations between childhood illnesses and NP microbiota. Our findings suggest that early-life colonization with *S*. *pneumoniae*, *H*. *influenzae*, *and M*. *catarrhalis* is associated with increased occurrence of clinically significant respiratory conditions in early life.

## Results

### Child cohort and analysis of physician-attended illnesses

Pediatric physician-attended illnesses were prospectively recorded in the medical record in a cohort of 358 children from Rochester, NY (Table 1). For viral URIs when parents brought the child for care based on their level of concern, a physician diagnosis was made based on pre-established criteria [9–12] and confirmed by PCR detection of a respiratory virus [12] (see Methods). Two-thirds of all physician-attended illness visits in the study occurred in the 6–30 month age window (S1 Fig).

**Table 1:** Study cohort demographics.

Variable	All Children (n = 358)		
	Number	%	n.r.
Sex		0.9	3
*Male*	197	55	
*Female*	158	44.1	
Race		0	0
*White*	284	79.3	
*African-American*	48	13.4	
*Mixed/Other*	26	7.3	
Siblings		8.7	31
*Yes*	203	56.7	
*No*	124	34.6	
Daycare		11.5	41
*Yes*	126	35.2	
*No*	191	53.4	
Feeding		10.1	36
*Breastfed*	46	12.8	
*Formula*	160	44.7	
*Both*	116	32.4	
Smoking in home		8.7	31
*Yes*	30	8.4	
*No*	297	83	
Atopy		11.5	41
*Yes*	110	30.7	
*No*	207	57.8	

n.r = not reported.

**Fig 1:**
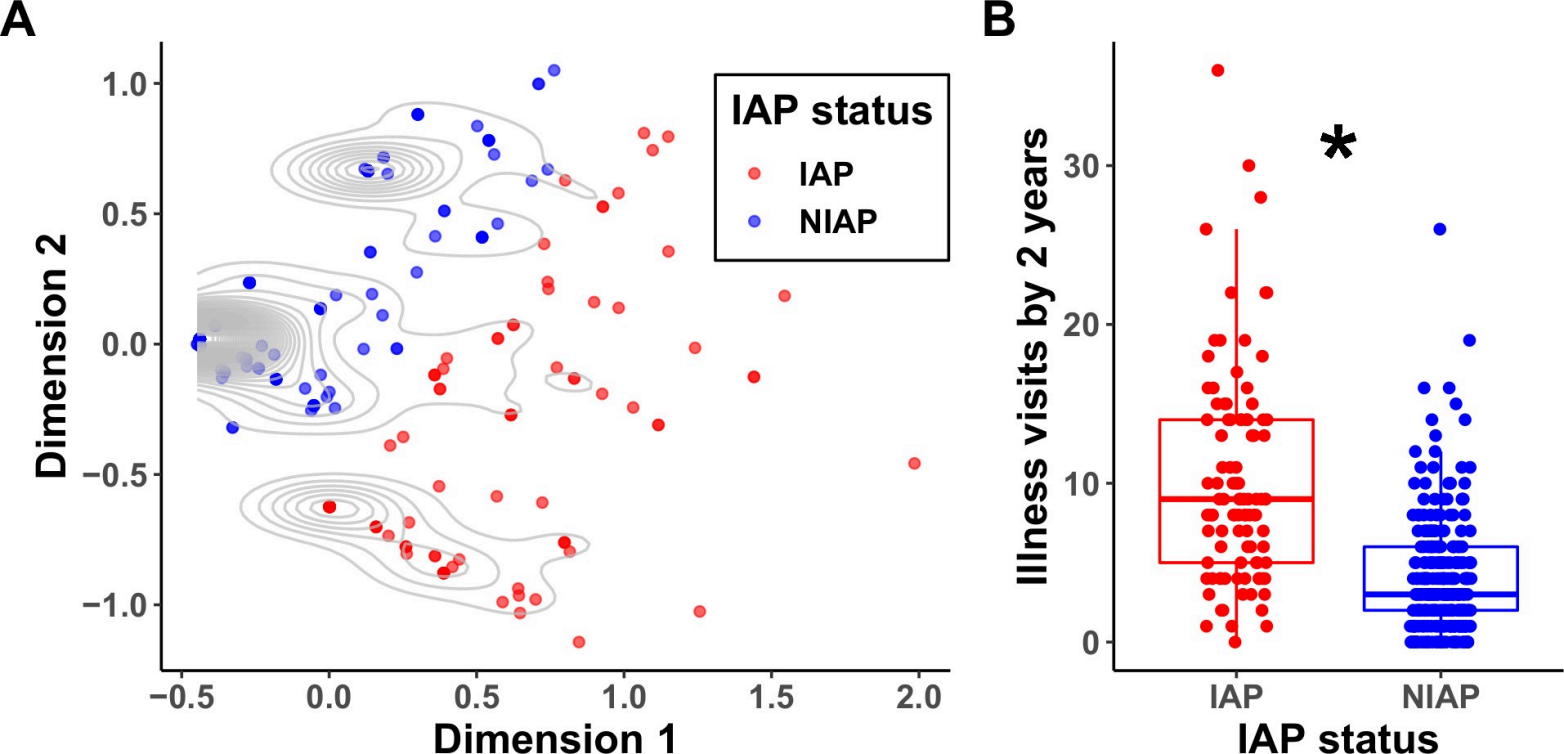
Identification of infection and allergy-prone (IAP) children. A) Identified clusters from cohort of 358 children based on physician diagnosis of illnesses from 6 to 60 months of age. Contours show the density of subjects independent of assigned IAP status. B) Comparison of overall physician-attended illness visits from 6 to 30 months of age in IAP (red, n = 99) and NIAP (blue, n = 259) children. *: p value <0.05 by Wilcox test.

In order to define sub-groups of children within the cohort, an unsupervised clustering analysis was used. The cohort successfully clustered into two groups, showing marked differences in the prevalence of respiratory infections, asthma, and allergy (Fig 1A). We termed the two groups infection and allergy prone (IAP) and non-infection and allergy prone (NIAP). IAP children had significantly more physician-attended illness visits from 6–30 months of age compared to NIAP children, corroborating the clustering analysis (Fig 1B). In a comparison of demographic and risk factors between the two groups, the proportion of children that were male, were non-breastfed, or attended daycare were higher in the IAP group (S1 Table). In a multivariable analysis to determine which demographic/risk factors contributed most to the IAP phenotype, male gender and daycare attendance were statistically significant (S2 Table), along with marginal significant evidence that formula feeding increased the risk of IAP status. These findings are consistent with previous reports [13].

**Fig 2:**
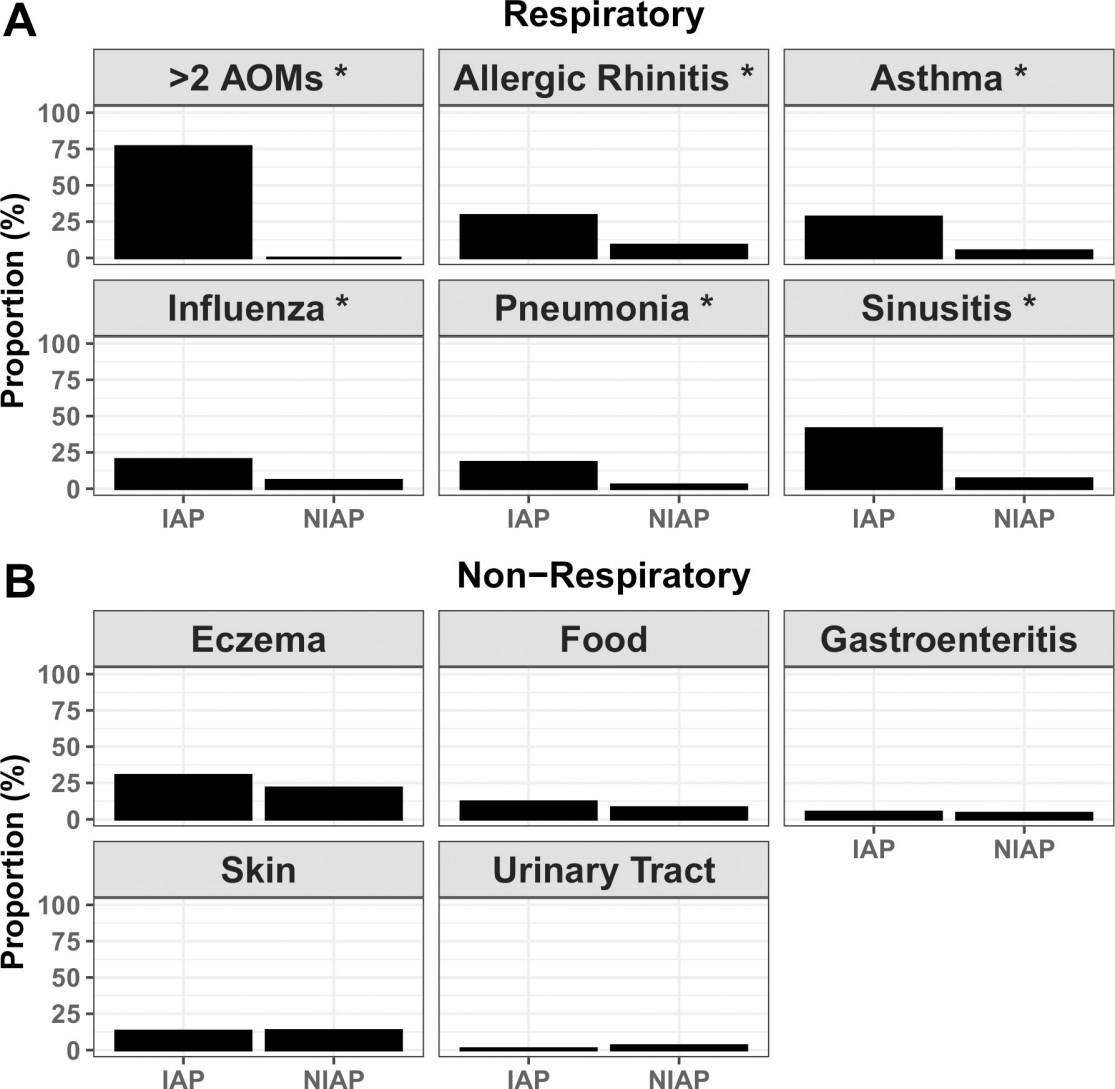
Comparison of individual conditions in IAP and NIAP children. Physician diagnosis of various respiratory (A) and non-respiratory (B) conditions were compared between IAP and NIAP children. *: p value <0.05 by chi square test comparing proportions.

In further analysis of the type of infections reported, IAP children were significantly more likely to experience recurrent AOM, influenza, sinusitis, pneumonia, asthma, and allergic rhinitis compared to NIAP children (Fig 2A). The two groups did not experience significantly different rates of eczema, food allergy, skin infections, urinary tract infections, or acute gastroenteritis (Fig 2B). It is important to note that, while urinary tract infection and acute gastroenteritis rates were not significantly different between the groups, the overall incidence of these conditions in the cohort was low (2.5% for urinary tract infection, 4.5% for acute gastroenteritis).

### Association between pathobiont colonization and IAP phenotype

**Fig 3:**
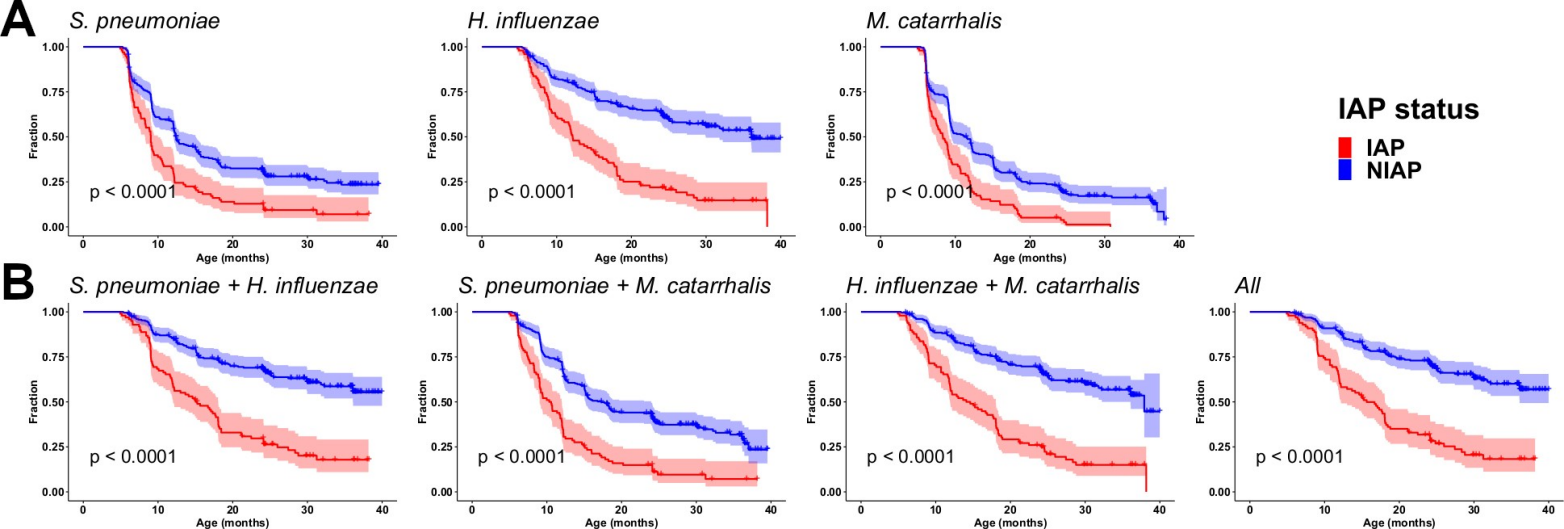
Pathobiont colonization in IAP and NIAP children. A) The age at first colonization with *S*. *pneumoniae*, *H*. *influenzae* and/or *M*. *catarrhalis* as microbiologically identified in child NP samples from 6 to 40 months of age are shown (n = 98 IAP, 256 NIAP). The proportion of colonization-negative children throughout the time course are represented as the surviving cohort in the analysis. B) Age at first colonization with combinations of pathobionts as shown. p values from log-rank test are displayed on each graph.

Since respiratory infectious and allergic illnesses had a higher incidence in IAP children and many of these illnesses have been associated with the respiratory pathogens *S*. *pneumoniae*, *H*. *influenzae*, *and M*. *catarrhalis*, we hypothesized that early-life colonization of the nasopharynx (NP) with these bacteria may associate with the IAP profile. Therefore, colonization events with *S*. *pneumoniae*, *H*. *influenzae*, and *M*. *catarrhalis* that had been identified by microbiological culture in nasal swab samples during prospective collections at both healthy and illness visits from 6–36 months of age were analyzed for association with the IAP or NIAP phenotype. We found that age at first NP colonization with any of the three pathobionts was significantly associated with the IAP phenotype. Specifically, IAP children experienced colonization at an earlier age than NIAP children did for all 3 bacteria (Fig 3A). In an analysis of individual conditions, early *M*. *catarrhalis* colonization significantly associated with pneumonia, sinusitis, and asthma susceptibility, *H*. *influenzae* with pneumonia, sinusitis, influenza, and allergic rhinitis, and *S*. *pneumoniae* with sinusitis (S2 Fig). Additionally, there was an unexpected association observed between early age at first *S*. *pneumoniae* colonization and reduced incidence of food allergy (S2 Fig). Overall, *H*. *influenzae* was the most highly segregated pathobiont between IAP and NIAP children in this analysis. We further analyzed pathobiont colonization data for co-colonization patterns in IAP and NIAP children. We found that co-colonization with any combination of the three pathobionts in early life was highly predictive of IAP phenotype development (Fig 3B). The difference between the median age at first colonization by multiple pathobionts in IAP vs NIAP children was greater for all combinations (*S*. *pneumoniae*+*H*. *influenzae*, *S*. *pneumoniae*+*M*. *catarrhalis*, *H*. *influenzae*+*M*. *catarrhalis*, or all 3 pathobionts) than for any individual species. Approximately 50% of IAP children experienced colonization by all 3 pathobionts by 15.6 months of age, while only 34% of NIAP children were colonized by 40 months of age. IAP status was associated with increased hazard ratios for early colonization by each pathobiont either individually or in combination (Table 2). All of these increases remained significant after adjusting for possible contributions from other demographic variables (Table 3). These data suggest that early life NP colonization with pathobionts is associated with subsequent increased risk of an array of respiratory illnesses in the first years of life.

**Table 2:** Unadjusted Cox proportional hazard models for NP colonization.

Colonization	Coefficient	Hazard ratio	Conf. interval	p value
*S*. *pneumoniae*	0.651	1.92	1.49–2.47	5.10E-07
*H*. *influenzae*	1.116	3.05	2.29–4.08	3.90E-14
*M*. *catarrhalis*	0.675	1.96	1.54–2.51	6.26E-08
*S*. *pneumoniae+ H*. *influenzae*	1.144	3.14	2.32–4.25	1.55E-13
*S*. *pneumoniae+ M*. *catarrhalis*	0.887	2.43	1.87–3.16	3.21E-11
*H*. *influenzae+ M*. *catarrhalis*	1.24	3.46	2.57–4.65	2.63E-16
*All*	1.232	3.43	2.51–4.67	6.66E-15

95% confidence intervals and p values are as shown.

**Table 3:** Cox proportional hazard models for NP colonization adjusted for demographic variables.

Colonization	Coefficient	Hazard ratio	Conf. interval	p value
*S*. *pneumoniae*	0.534	1.71	1.27–2.30	4.36E-04
*H*. *influenzae*	0.901	2.46	1.76–3.44	1.41E-07
*M*. *catarrhalis*	0.496	1.64	1.25–2.16	4.26E-04
*S*. *pneumoniae+ H*. *influenzae*	0.964	2.62	1.85–3.73	7.66E-08
*S*. *pneumoniae+ M*. *catarrhalis*	0.709	2.03	1.50–2.75	4.25E-06
*H*. *influenzae+ M*. *catarrhalis*	1.045	2.84	2.02–4.00	2.09E-09
*All*	1.066	2.9	2.03–4.15	5.23E-09

95% confidence intervals and p values are as shown.

In order to determine whether specific viruses affected the likelihood of nasopharyngeal colonization by bacterial pathobionts, PCR was used to detect 10 different respiratory viruses in NP swabs at 236 illness visits from 162 children. At least one virus was detected in 156 samples (66.1%). Overall, detection of any virus with any otopathogen in illness visit samples were not significantly associated, suggesting the two were not dependent on each other (S3 Table). In a further analysis of the association of specific viruses and otopathogens, we found a statistically significant association between RSV infection and *H*. *influenzae* colonization (S4 Table). All other virus-otopathogen combinations were not significantly associated. There were insufficient data to model associations between viral infection and subsequent pathobiont colonization as visits were usually three months apart.

### NP microbiome analysis

**Fig 4:**
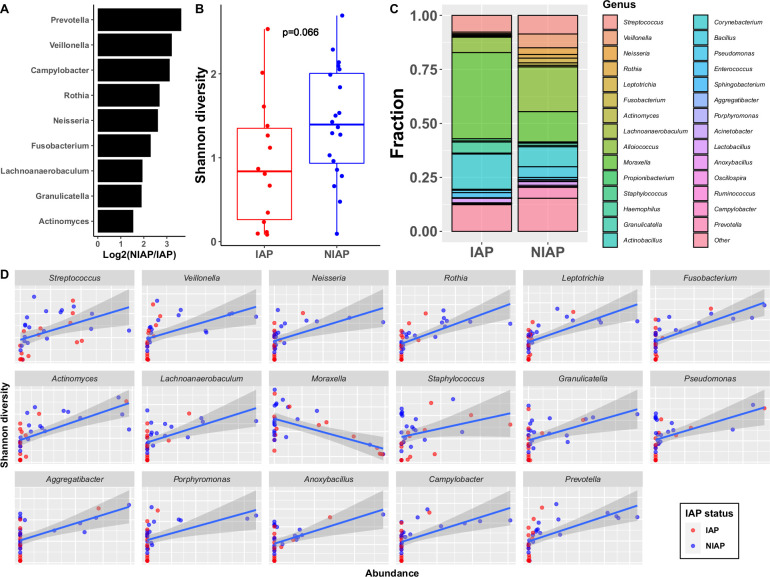
Nasopharyngeal 16S rRNA analysis at 6 months of age. A) Genera found to be differently abundant between IAP and NIAP groups by pairwise Wilcox with Holm-Bonferroni adjustment. B) Shannon diversity of NP microbiome in IAP and NIAP children at 6 months of age. p value for Wilcox test is shown. C) Average frequency of each genus in IAP and NIAP children. D) Correlation analysis of individual genus frequency and Shannon diversity for each child. Shown are all genera that were significantly correlated (p<0.05) using a linear model expressing Shannon diversity as a function of each bacterial genus and IAP status. n = 14 IAP and 20 NIAP samples analyzed.

Since early colonization with pathobionts was strongly associated with respiratory illnesses in our cohort, for the purpose of this study NP microbiota analysis using 16S rRNA sequencing was performed on an available subset of samples. Nasal wash samples from well-child visits at the first sampling time point (6 months of age) were characterized, since this time point was the beginning of the susceptibility window for respiratory infections in our cohort (S1 Fig). In 14 IAP and 20 NIAP children, 29 genera were identified with >1% relative abundance in at least one sample. Lower abundance genera were excluded from further analysis. Nine genera were found to be differentially abundant comparing IAP and NIAP children (Fig 4A and S5 Table). Shannon diversity trended lower in IAP children (Fig 4B; p = 0.066). *Moraxella* was the most abundant genus recovered in the population overall (Fig 4C). Among IAP subjects, *Moraxella* was significantly more abundant than 25 of the 28 other genera (excepting only *Streptococcus*, *Alloiococcus*, and *Corynebacterium*), while among NIAP subjects, *Moraxella* was significantly more abundant than 17 of the 28 other genera (p<0.05 by Wilcoxon test). No genus was significantly more abundant than *Moraxella* in either NIAP or IAP subjects. In a correlation analysis of genus abundance and sample diversity, the abundance of 17 out of 29 genera significantly correlated with Shannon diversity. However, *Moraxella* was the only genus that negatively correlated with Shannon diversity (Fig 4D). In an analysis of pathobiont colonization status and NP microbiota, NP colonization by otopathogens did not significantly impact the abundance of any commensal genera in the NP (S6 Table).

## Discussion

Our study reports three important findings: 1) children can be clustered into two groups with reference to respiratory infection and allergic proneness; 2) early-life NP colonization with *S*. *pneumoniae*, *H*. *influenzae*, and *M*. *catarrhalis* associates with IAP children; and 3) *Moraxella* abundance negatively associates with NP microbiota diversity measured at 6 months of age.

We undertook this retrospective analysis of samples collected from our prospectively-enrolled child cohort with prior knowledge that the children segregated into a sub-population that was prone to otitis media and a non-prone group [5, 14]. We had previously shown that children prone to otitis media have an increased incidence of atopy [14], often failed to respond to routine vaccinations [15], and had multiple deficiencies in innate and adaptive immunity [16]. Those findings provided the premise that a child phenotype might exist where infection and atopic illnesses occurred significantly more frequently. The unbiased clustering analysis supported the premise that children clustered into respiratory infectious and allergic prone and non-prone groups. This was not strictly driven by the frequency of occurrence of a given condition within the cohort, since eczema had the highest overall occurrence of all conditions but was not enriched in the IAP group. The reliability of the clinical record for the cohort adds strength to this result, with <2% missing data for the conditions studied (see Methods).

It is known that bacterial interactions between NP pathobionts can enhance bacterial fitness in the host. In a child study in Greenland, *H*. *influenzae* and *S*. *pneumoniae* NP colonization were both significantly associated with *M*. *catarrhalis* co-colonization [17]. In our previous work and others, *M*. *catarrhalis* was found to have a high NP co-colonization rate with other pathobionts but was rarely isolated from the middle ear during acute otitis media compared to *H*. *influenzae* and *S*. *pneumoniae* [18–20]. In addition, NP co-colonization of *M*. *catarrhalis* with *S*. *pneumoniae* or *H*. *influenzae* increased the risk of progression to AOM [21]. A synergy also exists between *H*. *influenzae* and *M*. *catarrhalis* in that co-colonization enhances biofilm formation and antibiotic resistance compared to mono-colonization in a culture system [22]. Although competition also occurs between pathobionts, these data suggest that co-colonization can facilitate niche establishment, and further that the presence of *M*. *catarrhalis* may increase *H*. *influenzae* and *S*. *pneumoniae* virulence. We found that NP colonization with *Moraxella* at 6 months of age was associated with reduction in overall microbiota diversity, co-colonization with *H*. *influenzae* and *S*. *pneumoniae*, and increased risk of respiratory illnesses. Published work from Teo, *et*. *al*. further linked infant NP microbiome with severity of respiratory infections and development of asthma [6]. Since pathobiont interactions appear to be largely synergistic in the NP, a vaccine against one may be additionally beneficial in reducing the disease-causing burden of others [23]. While the PCV13 vaccine has significantly reduced pneumococcal disease from vaccine serotypes since its inception, non-vaccine serotypes are on the rise and demand the development of novel vaccine strategies for protection from *S*. *pneumoniae* [24]. These vaccines may have a greater benefit than expected of reducing the prevalence of respiratory conditions that co-occur in some children.

Given that several respiratory infections and asthma have been associated with pathobiont exposure, it is clear that therapies addressing these pathogens are of considerable importance. Aside from vaccine development, probiotic therapy is one approach that may result in not only promoting microbial diversity, but also increasing competition with pathobionts. Indeed, clinical trials are already ongoing for therapeutic introduction of probiotics to the respiratory tract. Our data suggests that interactions between pathobionts is an important factor in promoting niche competition and effectively ‘bullying’ other commensals from the NP space. While no specific commensal genus seemed to promote reduced pathobiont colonization in our analysis, this microbiome work is still in early stages and analyses of commensals at the species level over time is underway to identify potential novel probiotic candidates for pathobiont control. For instance, one study suggested NP *Staphylococcus* and *Sphingobium* abundance may limit AOM complication during URI [25]. Approaches targeting pathobionts and commensals are both important and needed to address susceptibility to multiple respiratory conditions.

Since children often ‘grow out’ of their susceptibility to infections and converge to a more canonical phenotype by school age, an important question that arises is how microbiome development matures and stabilizes in the first years of life in relation to infection susceptibility. There is now convincing evidence that microbiome development occurs primarily in the post-natal environment, where pathobionts and commensals are competing for space [26, 27]. This process may take up to three years [7] and is influenced by infection, antibiotic use, *etc*. For instance, RSV infection has been shown to associate with increased colonization density of *Haemophilus* and *Streptococcus* species in the NP [28]. Our data suggest a similar synergy between RSV and *H*. *influenzae*, and other associations may be identified with larger data sets. In a mouse model of *Mycobacterium* infection, antibiotics-induced dysbiosis prior to infection resulted in earlier establishment of pathogenic lower respiratory tract infection and reduced anti-*Mycobacterial* immunity [29, 30]. In a longitudinal analysis of NP microbiota in the first two years of life, microbial profiles at six weeks of age were associated with variable stability in the NP microbiota profile in the first two years of life, with a more stable profile associating with lower incidence of upper respiratory infection [31]. These data all suggest that the NP ecosystem in early life is dynamic and shaped by environmental encounters that could sequentially contribute to resistance or susceptibility to immune and infectious conditions.

A limitation of this study is that the child cohort was enriched for children susceptible to recurrent AOM [14]. Therefore, the proportion of IAP children identified here is likely an overestimate of the actual frequency present in the US. For microbiota analysis, we had insufficient samples to more clearly define differences in the NP microbiome comparing IAP and NIAP children. In future work, systematic sampling starting after birth will yield a robust data set for mechanistic studies. While viral infection data showed an association between RSV infection and *H*. *influenzae* colonization, not all children in the cohort were tested for viral identification. Therefore, it is possible that virus-driven outcomes in infection-prone children may be underestimated in our study.

Taken together, these data add to the growing literature defining associations between NP microbiome and respiratory health, and further confirm key pathobionts involved in susceptibility. Identification of NP co-colonization with pathobionts in early life may be a non-invasive and effective diagnostic in determining children who will likely progress to the IAP phenotype. A confounding factor in studying IAP children is that this cohort, by definition, also receives the most antibiotics prescriptions. More work is needed to determine the potential effects of antibiotics in pathobiont colonization and infection susceptibility.

## Methods

### Study population

This study derives from a retrospective analysis of clinical samples collected from a prospectively-enrolled cohort of 358 children as well as new 16S sequencing and viral PCR analyses from NP samples. The primary objective of the primary prospective study from which the study cohorts were derived was to evaluate the occurrence of acute otitis media, including the instigating pathogens and immune response of otitis prone children. Our group has previously published on those results (summarized in refs [5, 14, 16]). A secondary objective of the prospective study not previously reported was to study all clinic-attended illness visits and their relationship to respiratory pathogen NP colonization. The current analysis is the first to address this secondary objective. From the medical record, all medical encounters that occurred from 6 to 60 months of age were analyzed for this study. As part of the prospective study design, NP swabs were collected at regularly-scheduled visits at 6 (n = 267 samples; 91 missing), 9 (315; 43 missing), 12 (319; 39 missing), 15 (292; 66 missing), 18 (302; 56 missing), 24 (300; 58 missing), and 36 (184; 174 missing) months of age with or without concurrent URI symptoms, and during AOM episodes (n = 484) and at AOM follow-up visits (154) throughout the duration of the study. Those swabs were tested for the presence of *S*. *pneumoniae*, *H*. *influenza*e, and *M*. *catarrhalis*. The children were from a middle class community in Rochester, NY. Virtually all medical care was provided at a single clinical practice site of one of the authors (MEP). In the community, children receiving care at any other regional site (*e*.*g*. urgent care or emergency room) had the details of their medical encounters shared electronically with the primary study site. Electronic medical records were available and included in the analysis for the enrolled cohort from birth to age 5 years. From those records, we determined that 95% percent of all illness visits occurred between 6 and 60 months of age. Illness visits before 6 months of age occurred in only 5% of children, and therefore our analysis started at the 6-month time point. Illnesses that were of insufficient concern to parents to seek out medical care were not included in the analysis (*i*.*e*. telephone calls seeking advice). Demographic and infection risk factor data collected included sex, race, siblings, breastfeeding, daycare attendance, tobacco smoke exposure, and atopy. The Rochester General Hospital IRB approved the study and written informed consent was obtained from parents before enrollment. All methods were performed in accordance with the IRB's relevant guidelines and regulations.

### Definitions

*Viral URI*: Physician clinical diagnosis of viral URI at visits was made when children presented with nasal congestion, rhinorrhea, cough, and/or sore throat with or without fever as previously established [9–12]. *AOM*: AOM was diagnosed using pneumatic otoscopy by validated otoscopists, when children with acute onset of symptoms consistent with AOM had tympanic membranes (TMs) that were: 1) bulging or full, and 2) a cloudy or purulent effusion was observed, or the TM was completely opacified, and 3) TM mobility was reduced or absent according to American Academy of Pediatrics guidelines [32]. Recurrent AOM was defined as three or more episodes of AOM up to age five. *Lobar pneumonia and sinusitis*: lobar pneumonia and sinusitis were clinically diagnosed based on Infectious Diseases Society of America and American Academy of Pediatrics (AAP) guidelines, respectively [33, 34]. *Influenza*: Influenza virus was diagnosed clinically and by rapid test detection of the virus by NP swab. *Asthma*, *food allergy*, *allergic rhinitis*, *eczema*: Diagnosis of asthma, food allergy, allergic rhinitis, and eczema followed the AAP and American Academy of Allergy, Asthma, and Immunology guidelines [35–37], and were confirmed by a board-certified pediatric allergist. *Skin infections*, *acute gastroenteritis*, *and urinary tract infection*: prospective definitions of skin infection, acute gastroenteritis, and urinary tract infection were not in the study protocol; therefore, the diagnosis as recorded in the medical record was deemed accurate. These conditions occurred in the children as follows (condition: # of children diagnosed, # not reported): pneumonia: 25, 0; influenza: 35, 0; urinary tract infection: 9, 0; acute gastroenteritis: 16, 0; skin infection: 48, 1; asthma: 41, 1; food allergy: 33, 1; allergic rhinitis: 52, 0; eczema: 86, 0; sinusitis: 59, 6; recurrent AOM: 82, 0.

### Bacterial 16S rRNA sequencing

Nasal wash samples from 35 children at 6 months of age were collected in PBS and then immediately frozen at -80°C. They were sent to the Microbiome Core Facility at University of North Carolina (https://www.med.unc.edu/microbiome) for DNA extraction and sequencing. A negative control (DNA/RNA-free water) and a positive control (a defined community containing eight bacteria and two yeast, Zymo Research, Irvine, CA) were processed with the samples to ensure quality of the runs. DNA was extracted using the KingFisher™ Flex Purification System and the V4 region of the bacterial 16S rRNA gene was amplified. Primer sequences were: 515F - 5’ TCGTCGGCAGCGTCAGATGTGTATAAGAGACAGGTGCCAGCMGCCGCGGTAA 3’; 806R - 5’GTCTCGTGGGCTCGGAGATGTGTATAAGAGACAGGGACTACHVGGGTWTCTAAT 3’. Sequencing was performed using Illumina MiSeq and converted to fastq format and demultiplexed using Illumina Bcl2Fastq 2.18.0.12. The resulting paired-end reads were processed, and index and linker primer sequences trimmed using cutadapt in QIIME 2. The reads were processed with default settings in DADA2 to merge paired ends, perform quality filter, error correction, and removal of chimera. Amplicon sequencing units from DADA2 were assigned taxonomic identifiers at 97% identity with respect to Green Genes release 13_08 using the QIIME 2 q2-featureclassifier detection [38]. The OTU table created was imported to Excel for calculation of DNA reads and for converting total reads to percentage. Samples with total DNA reads fewer than 1000 were excluded, leaving a total of 34 samples qualified for further analyses. OTUs with more than 1% relative abundance in at least one of the samples were kept for further analyses.

### Microbiology

NP colonization events with *M*. *catarrhalis*, *S*. *pneumoniae*, and *H*. *influenzae* were identified in nasal swab and nasal wash samples during both healthy and illness visits from children 6–36 months of age. The protocol for microbiological identification of these pathogens was as previously described [12, 39] Briefly, samples were plated on Chocolate II agar and tryptic soy agar (TSA) with 5% sheep blood (BD). Colonies were then selected for further analysis. *S*. *pneumoniae*: colony morphology, alpha-hemolysis on TSA plates, and inhibition by optochin were used to confirm species. *H*. *influenzae*: colony morphology, gram stain, growth combined with lack of hemolysis in quadrant IV of QuadID plate, and inability to synthesize porphyrin were used for identification. *M*. *catarrhalis*: colony morphology, gram stain, oxidase reaction, and reactivity to catarrhalis disk (Remel). In each case, sub-culturing was done to confirm isolate.

### Virus detection

Real-time PCR was used to identify presence of influenza A virus, enterovirus, adenovirus B and C, parainfluenza virus III, rhinovirus, RSV, bocavirus, metapneumovirus, and seasonal coronavirus in NP samples when URI symptoms were present. Briefly, RNA was isolated from NP swab samples in viral transfer media using Trizol method. Primers for viral detection, positive controls, and reagents were purchased from Genesig (Primer Design). Universal probe sets from Biorad were used. PCR reactions were performed on an iCycler (Biorad).

### Infection proneness criteria

Experiences of pneumonia, influenza, urinary tract infection, skin infection, sinusitis, acute gastroenteritis, asthma, food allergy, environmental allergy, eczema, and recurrent AOM (at least 3 episodes) were expressed as binary criteria, creating a vector of illness conditions for each subject. Multidimensional scaling (MDS) was performed on the pairwise Euclidean distance matrix for these subject vectors to yield two dimensions. Unsupervised K-means clustering was then applied to the MDS dimensions to identify two clusters of subjects. Mapping these subjects onto the original illness criteria and an independent record of illness visits per subject showed that one cluster was highly enriched for both individual respiratory illnesses and illness visits overall. These analyses were performed using functions in base R version 3.6.1 [40].

### Other statistics

The prevalence of each illness condition in IAP and NIAP groups was compared using chi-squared tests. Ages at first colonization by pathobionts were compared using log-rank tests and Cox proportional hazard models with IAP/NIAP status or the various individual illness conditions as the grouping factors using functions from the survival [41, 42] and survminer [43] packages in R. Univariate risk factors associated with IAP status were compared using chi-squared tests, with multivariable risk factors tested by logistic regression. The effect of otopathogen colonization on the abundance of each commensal genus was assessed by Wilcoxon test, using the Benjamini-Hochberg correction for multiple comparisons. Pairwise Wilcox test with Holm-Bonferroni adjustment were used for the 16S sequencing composition analysis.

## Supporting information

S1 Fig Total physician-attended illness visits in 358 children from 6 to 60 months of age. (DOCX)

S2 Fig Relationship between age at first colonization with pathobionts and physician-diagnosed conditions. (DOCX)

S1 Table Cohort demographics table, with IAP and NIAP children compared. (DOCX)

S2 Table Multivariable regression of demographics with odds ratios and confidence intervals. (DOCX)

S3 Table Overall detection of viruses and otopathogens in the subset of illness visits. (DOCX)

S4 Table Relationship between otopathogen colonization and detected respiratory viruses at illness visits. (DOCX)

S5 Table Significantly different genera comparing the NP microbiome of IAP and NIAP groups. (DOCX)

S6 Table Relationship between otopathogen colonization status and commensal genera identified by 16S sequencing. (DOCX)
