# Supplementary material for: Nasopharyngeal colonization with pathobionts is associated with susceptibility to respiratory illnesses in young children
Source: PLoS One. 2020 Dec 11;15(12):e0243942. doi: 10.1371/journal.pone.0243942 (PMC7732056; doi:10.1371/journal.pone.0243942)

S1 Fig. Total physician-attended illness visits in 358 children from 6 to 60 months of age. 66% of all visits reported occurred from 6 to 30 months of age.


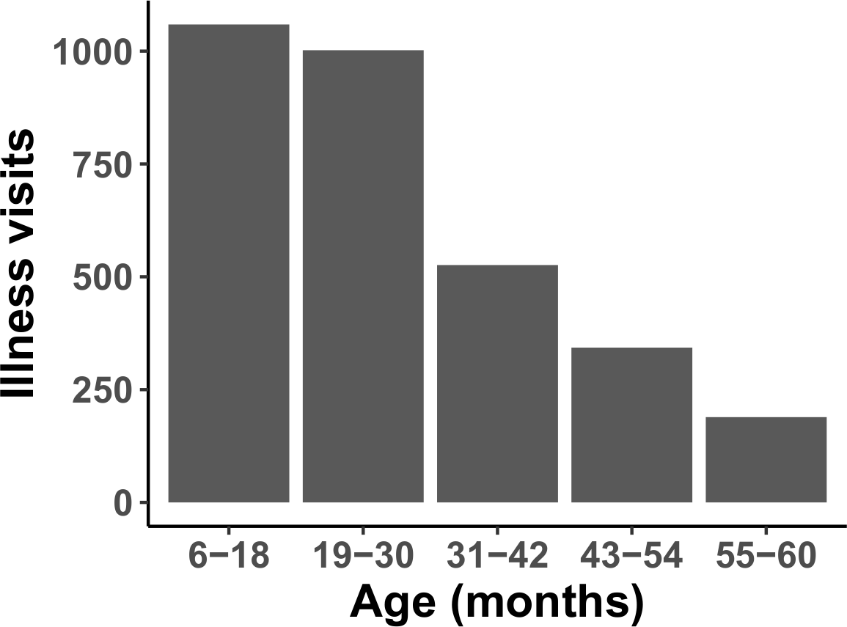

Supplement: S1 Fig — (DOCX) [file pone.0243942.s001.docx]
