# Supplementary material for: Nasopharyngeal colonization with pathobionts is associated with susceptibility to respiratory illnesses in young children
Source: PLoS One. 2020 Dec 11;15(12):e0243942. doi: 10.1371/journal.pone.0243942 (PMC7732056; doi:10.1371/journal.pone.0243942)

S2 Fig. Relationship between age at first colonization with pathobionts and physician-diagnosed conditions. Columns represents each of the three studied pathobionts: *S. pneumoniae*, *H. influenzae*, and *M. catarrhalis*. Each row is a respiratory (A) or non-respiratory (B) condition. p values for log rank test are shown on each graph.


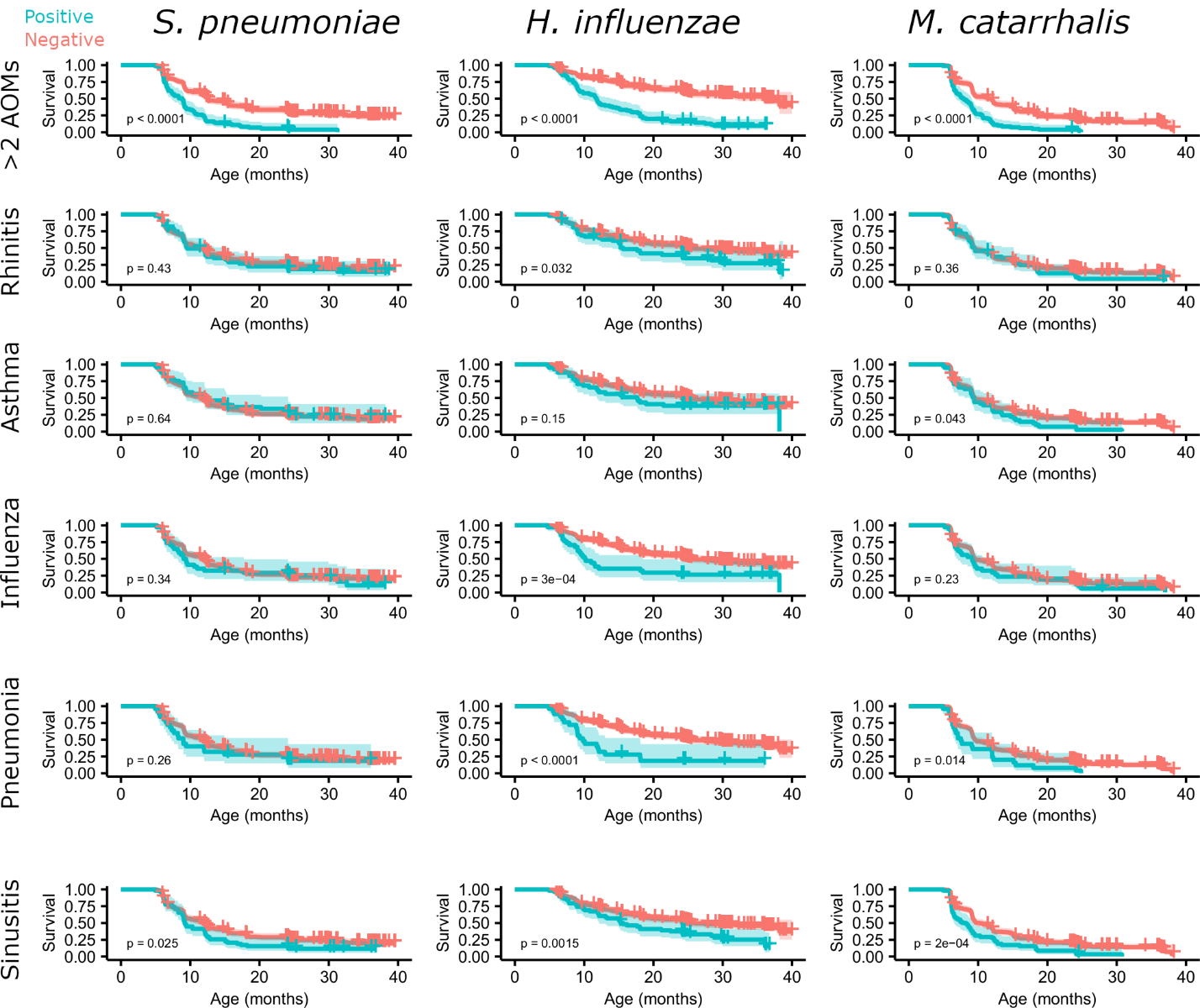


**A**

**B**


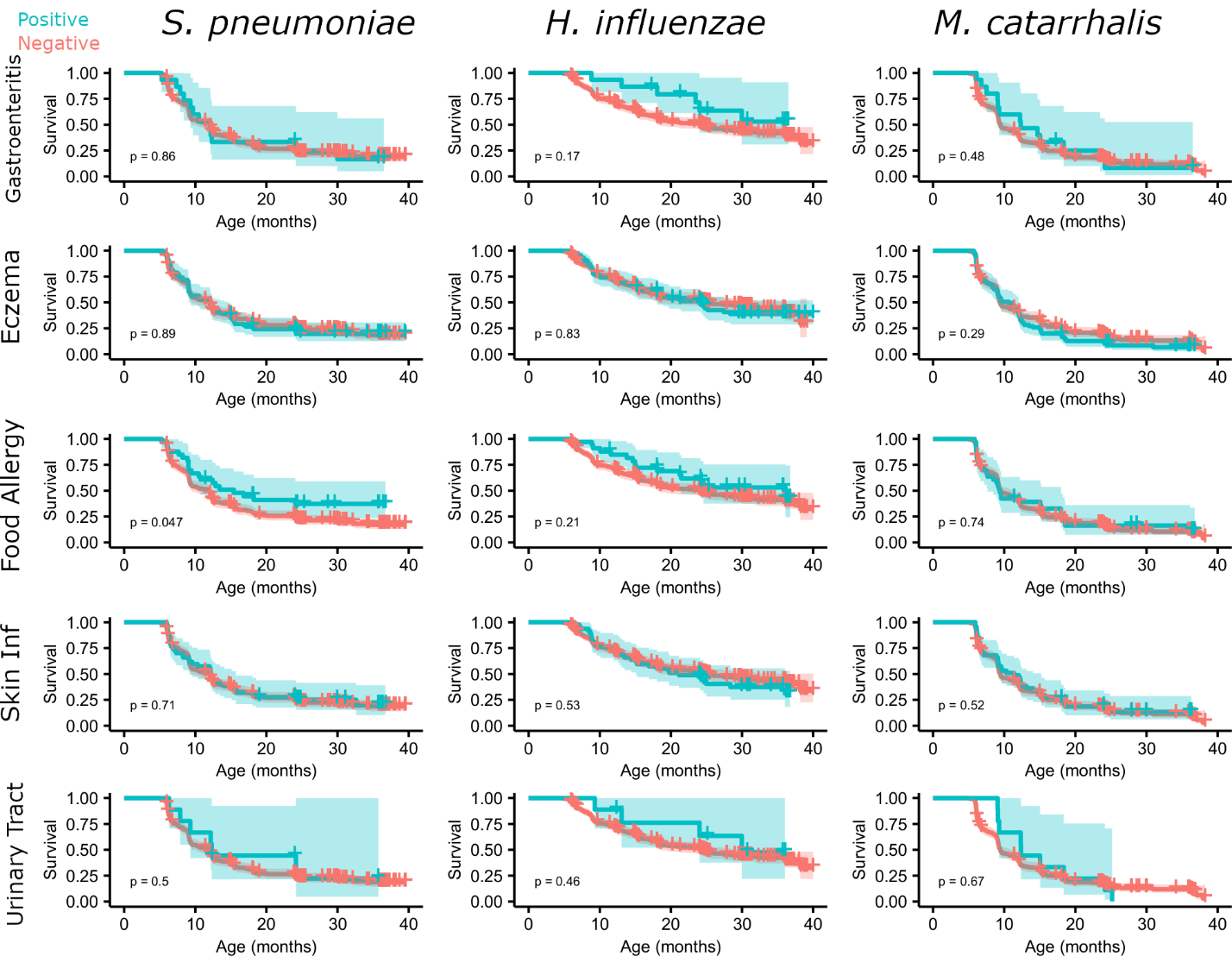

Supplement: S2 Fig — (DOCX) [file pone.0243942.s002.docx]
