# Supplementary material for: Nasopharyngeal colonization with pathobionts is associated with susceptibility to respiratory illnesses in young children
Source: PLoS One. 2020 Dec 11;15(12):e0243942. doi: 10.1371/journal.pone.0243942 (PMC7732056; doi:10.1371/journal.pone.0243942)
Supplement: S1 Table — (DOCX) [file pone.0243942.s003.docx]

S1 Table. Cohort demographics table, with IAP and NIAP children compared. Number of children in each group are under IAP and NIAP headings. IAP (%) and NIAP (%) are the proportion of children in this group. p values represent results of chi squared tests for each demographic variable to compare proportions.
