# Supplementary material for: Nasopharyngeal colonization with pathobionts is associated with susceptibility to respiratory illnesses in young children
Source: PLoS One. 2020 Dec 11;15(12):e0243942. doi: 10.1371/journal.pone.0243942 (PMC7732056; doi:10.1371/journal.pone.0243942)
Supplement: S2 Table — (DOCX) [file pone.0243942.s004.docx]

S2 Table. Multivariable regression of demographics with odds ratios and confidence intervals. In a logistic regression, IAP status was expressed as a function of sex, race, presence of siblings, daycare attendance, breastfeeding, and smoke exposure with all two-way interactions. Factors with the highest likelihood of impacting IAP development are shown.
