# Supplementary material for: Nasopharyngeal colonization with pathobionts is associated with susceptibility to respiratory illnesses in young children
Source: PLoS One. 2020 Dec 11;15(12):e0243942. doi: 10.1371/journal.pone.0243942 (PMC7732056; doi:10.1371/journal.pone.0243942)
Supplement: S3 Table — (DOCX) [file pone.0243942.s005.docx]

S3 Table. Overall detection of viruses and otopathogens in the subset of illness visits. Proportions of samples testing positive for any respiratory viruses and/or bacterial otopathogens were compared by chi-square test.
