# Supplementary material for: Nasopharyngeal colonization with pathobionts is associated with susceptibility to respiratory illnesses in young children
Source: PLoS One. 2020 Dec 11;15(12):e0243942. doi: 10.1371/journal.pone.0243942 (PMC7732056; doi:10.1371/journal.pone.0243942)
Supplement: S4 Table — (DOCX) [file pone.0243942.s006.docx]

S4 Table. Relationship between otopathogen colonization and detected respiratory viruses at illness visits. Proportions of samples testing positive for the indicated respiratory viruses and/or bacterial otopathogens were compared by chi-square test.
