# Supplementary material for: Nasopharyngeal colonization with pathobionts is associated with susceptibility to respiratory illnesses in young children
Source: PLoS One. 2020 Dec 11;15(12):e0243942. doi: 10.1371/journal.pone.0243942 (PMC7732056; doi:10.1371/journal.pone.0243942)
Supplement: S5 Table — (DOCX) [file pone.0243942.s007.docx]

S5 Table. Significantly different genera comparing the NP microbiome of IAP and NIAP groups.
