# Supplementary material for: Nasopharyngeal colonization with pathobionts is associated with susceptibility to respiratory illnesses in young children
Source: PLoS One. 2020 Dec 11;15(12):e0243942. doi: 10.1371/journal.pone.0243942 (PMC7732056; doi:10.1371/journal.pone.0243942)
Supplement: S6 Table — (DOCX) [file pone.0243942.s008.docx]

S6 Table. Relationship between otopathogen colonization status and commensal genera identified by 16S sequencing. P values for each interaction are as shown. No relationships are significant after multiple comparisons adjustment (in parentheses).
